# Assessing sustainability on the modern Silk Road: An objective weighting methodological approach

**DOI:** 10.1371/journal.pone.0324538

**Published:** 2025-05-20

**Authors:** Chia-Nan Wang, Hoang-Kha Nguyen, Nhat-Luong Nhieu

**Affiliations:** 1 Department of Industrial Engineering and Management, National Kaohsiung University of Science and Technology, Kaohsiung, Taiwan; 2 College of Technology and Design, University of Economics Ho Chi Minh City, Ho Chi Minh City, Vietnam.; Al Muthanna University, IRAQ

## Abstract

The Silk Road Economic Belt (SREB), a major 21st-century initiative, aims to revive the historic Silk Road by connecting Asia, Europe, and Africa through a network of trade and cultural exchange routes. This study aims to assess sustainable development across sixteen countries situated in South Asia, West Asia, and Africa—regions that are central to the SREB but face diverse environmental and socio-economic challenges. To achieve this, a novel hybrid multi-criteria decision-making approach is proposed, combining the Method based on the Removal Effects of Criteria (MEREC) and Operational Competitiveness Ratings Analysis (OCRA). The MEREC method is used to determine objective weights for sustainability indicators by evaluating the impact of each criterion’s exclusion, while OCRA is employed to evaluate and rank countries based on both beneficial and non-beneficial indicators. The findings reveal significant disparities in sustainability performance across the studied countries. Israel ranked highest in sustainability, followed by Sri Lanka and Nepal, while India showed the lowest performance. These results provide valuable benchmarks and strategic insights for regional policy planning and sustainable development efforts within the SREB framework.

## Introduction

The Silk Road Economic Belt (SREB) represents one of the most ambitious and transformative global development initiatives of the 21st century [1]. It seeks to revive the legacy of the ancient Silk Road by fostering infrastructure, trade, and cultural connectivity across Asia, Europe, and Africa [2]. As part of this initiative, the modern Silk Road (MSR) aims to integrate these regions through an extensive network of transport corridors, seaports, economic zones, and development policies [3]. Among the regions most deeply involved in this network are South Asia, West Asia, and Africa—areas marked by rapid change, high potential for growth, and equally pressing sustainability concerns [4].

These regions are characterized by diverse geography, economic structures, and governance systems, yet they share a range of sustainability challenges. Common issues include the degradation of ecosystems, resource depletion, greenhouse gas emissions, socio-economic inequality, and the strain of rapid urbanization and industrialization. However, the nature and intensity of these challenges vary. South Asia struggles with agricultural vulnerability, dense population pressures, and climate-related risks [5,6]. West Asia is particularly affected by water scarcity, heavy dependence on fossil fuels, and geopolitical instability [7,8]. Meanwhile, African countries often face constraints related to institutional capacity, biodiversity loss, and access to clean energy and basic services [9,10]. These contextual differences underscore the need for both a comparative and regionally sensitive assessment framework [11,12]. The dual imperative of promoting economic growth while ensuring environmental preservation and social equity has made sustainability evaluation an essential dimension of the SREB’s long-term success [13,14]. To address this, a comprehensive and objective methodological framework is required—one that can capture multi-dimensional sustainability performance across countries with varied data quality, development levels, and policy environments.

This study introduces a hybrid Multi-Criteria Decision-Making (MCDM) approach that combines the Method based on the Removal Effects of Criteria (MEREC) and Operational Competitiveness Ratings Analysis (OCRA). This integration—referred to as the MEREC-OCRA method—was chosen over more traditional MCDM techniques such as the Analytic Hierarchy Process (AHP), Technique for Order Preference by Similarity to Ideal Solution (TOPSIS), and Data Envelopment Analysis (DEA) for several reasons. AHP is widely used but relies on subjective expert judgment, which may introduce biases and inconsistencies, particularly when evaluating sustainability across culturally and economically diverse regions [15]. TOPSIS, while useful for proximity-based ranking, often suffers from sensitivity to normalization and lacks a clear theoretical foundation for weighting criteria[16]. DEA, though powerful for efficiency analysis, requires large datasets and is limited when evaluating both beneficial and non-beneficial criteria simultaneously [17].

In contrast to traditional weighting methods that rely heavily on subjective expert opinions, the MEREC (Method based on the Removal Effects of Criteria) approach offers an objective, data-driven mechanism for determining the relative importance of evaluation criteria. Rather than requiring decision-makers to assign weights based on linguistic judgments or preference scales, MEREC systematically evaluates how the elimination of each criterion affects the overall performance of the alternatives under study. This marginal impact analysis ensures that criteria are weighted according to their actual influence on the decision-making process, thus eliminating the inconsistencies and biases that often accompany human judgment [18,19]. The objectivity and reproducibility of this technique make it particularly suitable for large-scale sustainability assessments, where transparency and analytical rigor are essential.

Complementing MEREC is the Operational Competitiveness Ratings Analysis (OCRA), which serves as a robust evaluation tool for assessing alternatives across both beneficial and non-beneficial indicators. OCRA utilizes a linear preference-based structure, enabling the clear and consistent interpretation of results, especially when dealing with heterogeneous datasets [20]. Its ability to process input (cost-type) and output (benefit-type) criteria independently allows for a more granular and realistic evaluation of each alternative’s performance. When combined, the MEREC-OCRA methodology offers a comprehensive hybrid framework that supports both objective weighting and structured ranking, bridging the gap between methodological rigor and practical usability. This integration ensures a balanced, transparent, and reproducible assessment of sustainability outcomes, making the approach well-suited to real-world decision-making contexts where data availability, quality, and consistency may vary significantly. Moreover, the adaptability of MEREC-OCRA across multiple domains and scales enhances its potential application for guiding evidence-based policy development in complex, multi-dimensional environments.

Accordingly, the primary objective pursued in this study is the application of the MEREC-OCRA method for the comprehensive assessment of sustainability across sixteen countries located within the geographical scope of the Silk Road Economic Belt (SREB), with particular attention given to the regions of South Asia, West Asia, and Africa. These regions, selected for their strategic significance and developmental complexity, are characterized by diverse environmental conditions, economic structures, and social dynamics. In order to capture the multi-faceted nature of sustainability, a total of fourteen quantitative indicators spanning three critical dimensions—environmental, economic, and social—have been identified and utilized in the evaluation process. Through the implementation of the MEREC-OCRA methodology, a systematic effort is made to determine the relative sustainability performance of each country by examining their strengths and weaknesses across the selected criteria. The objective weighting of indicators through MEREC, combined with the structured ranking capabilities of OCRA, facilitates an evidence-based understanding of how countries are progressing along various sustainability dimensions. The analysis is designed not only to highlight which countries perform better overall but also to uncover the key drivers that enable progress, as well as the critical bottlenecks that hinder development in specific national contexts. Moreover, the sustainability evaluation is aligned with the broader global agenda set forth by the United Nations Sustainable Development Goals (SDGs). By mapping the selected indicators to relevant SDG targets—such as those concerning economic productivity, environmental conservation, and social well-being—the study seeks to offer insights that are directly relevant to international development frameworks. The ultimate aim is to produce a set of policy-relevant findings that can support decision-makers, planners, and international development stakeholders in formulating effective strategies to enhance sustainability outcomes across the SREB, while maintaining sensitivity to the regional distinctions and challenges inherent in South Asia, West Asia, and Africa.

The structure of the paper is organized as follows: following this introduction, The Literature Review section reviews the relevant literature on sustainability evaluation and MCDM methods. The Methodology section details the MEREC-OCRA methodology. The Numerical Results and Sensitivity Analysis sections present the numerical analysis and country rankings. The Policy Recommendations section discusses the implications of the findings, and the Conclusion section concludes the study by outlining policy recommendations and future research directions.

## Literature review

### MCDM approaches and its applications

In this section, we present a comprehensive literature review on sustainable development priorities and the application of multi-criteria decision-making (MCDM) methods. In recent years, MCDM models have gained significant credibility and are increasingly employed across numerous domains, particularly those associated with sustainable development. Several studies have explored the application of MCDM approaches in sustainable development. Meanwhile, Sadaf et al. used the MCDM method to select suitable wave energy technology for sustainable development [21]. Mohammad et al. integrated DEMATEL, ANP, and MCDM methods to maximise sustainability indicators in agricultural photovoltaic-based renewable energy systems [22]. Similarly, Alkan applies the criteria importance through intercriteria correlation (CIRITIC), stepwise weight assessment ratio analysis (SWARA), and combined distance-based assessment (CODAS) methods to evaluate renewable energy systems towards sustainable development [23]. This research shows that the MCDM method can be changed to take into account how sustainable development is available and useful in different areas.

Further contributions to the application of MCDM in sustainable development have been made through literature reviews and innovative model integration. Brodny & Tutak conducted a systematic review of the use of MCDM to assess energy and climate sustainability in European Union member states, providing a broad overview of how these tools are applied across different projects and geographies [24]. In the Malaysian context, Li et al. used a fuzzy MCDM based on cumulative prospect theory to select a renewable energy development path for sustainable development. Narwane et al. used an integrated MCDM approach to formulate policies to effectively remove barriers to sustainable development of the biofuel industry in India [25].

Innovative model combinations and appropriate approaches also play an important role in expanding the use of MCDM in sustainable development. In their study, Karahan et al. combined MEREC and AROMAN to determine the level of sustainable competitiveness of Turkey compared to its neighbouring countries [26]. Bera & Satapathy used the OCRA methodology to analyse and rank the most important urban development challenges in Odisha’s urban renewal plan to achieve India’s sustainable development goals [27]. This suggests that new MCDM combinations can work well in this area. Wang et al. applied the integrated DEA (Data Envelopment Analysis) and MCDM model to evaluate global container shipping companies for sustainable development orientation, emphasising the usefulness of the model in evaluating potential investment outcomes [28]. Łuczak & Just used the MCDM approach with an optimal tail selection to assess the sustainable development of territorial units [29].

This analysis, founded on a robust and comprehensive framework for the prudent application of multi-criteria decision-making methods, provides an in-depth review of the existing body of literature on various approaches to assessing sustainable development. It focuses specifically on countries within the MSR region, offering a detailed exploration of the methodologies employed, their strengths and limitations, and the contexts in which they have been applied. The goal is to determine the weights of criteria and sub-criteria for evaluating sustainable development goals through a multi-criteria assessment methodology. The most commonly used methods for weighting in MCDM are presented in Table 1. In recent years, the MEREC method, along with its integration into hybrid approaches with other MCDM techniques, has gained significant traction in MCDM research due to its structured and objective analytical capabilities. This growing trend highlights its relevance in addressing complex decision-making challenges, particularly in fields like sustainable development. For this study, the selection of an appropriate model was guided by the need to address the multifaceted challenges of sustainable development assessment in the MSR region, where diverse economic, environmental, and social factors must be carefully balanced. The MEREC method was chosen for its ability to provide objective and unbiased criterion weighting, a feature that is critical in evaluating renewable energy projects, where decision-making requires reconciling technical reliability, environmental sustainability, economic feasibility, and social acceptability. Unlike other MCDM methods, such as AHP (Analytic Hierarchy Process) or fuzzy logic, which often depend on expert judgements or linguistic evaluations that can introduce subjective biases, MEREC employs an entropy-based approach to calculate weights. By systematically removing each criteria and analysing its impact on the overall decision-making process, MEREC ensures a fair and impartial weighting process, providing a transparent and credible framework. This objectivity enhances the reliability of the results and makes MEREC particularly well-suited for complex applications in sustainable development, where impartiality and methodological rigour are paramount.

**Table 1:** MCDM application for sustainable development problem.

No.	Author(s)	Year	CMDM Method	Other Method
1	Li et al. [30]	2020	TOPSIS & VIKOR	ANP
2	Aljaghoub et al. [31]	2022	TOPSIS	
3	Zhao et al. [32]	2022	TOPSIS	Entropy
4	Foroozesh et al. [33]	2022	AHP & TOPSIS	GIS
5	Ebad et al. [34]	2020	TOPSIS	
6	Saraswat & Digalwar [35]	2021	AHP	Entropy & Fuzzy
7	Kaya & Sema [36]	2020	MABAC & WASPAS	MAIRCA
8	Brodny & Tutak [37]	2023	CRITIC	Entropy
9	Paz et al. [38]	2021	TOPSIS	
10	Alimohammadlou & Khoshsepehr [39]	2022	AHP & WASPAS	
11	Bilgili et al. [40]	2022	IF-TOPSIS	
12	Liu et al. [41]	2022	TODIM-ERA	
13	Ecer [42]	2021	BWM	
14	Brodny & Tutak [43]	2021	TOPSIS-VIKOR-MOORA & COPRAS	
15	Solangi et al. [44]	2021	AHP & TOPSIS	

### The silk road economic belt related studies

The SREB, as a strategic component of the Belt and Road Initiative (BRI), has attracted considerable scholarly attention in recent years due to its implications for sustainable development, economic diplomacy, environmental governance, and regional cooperation. Several empirical and theoretical studies have explored diverse aspects of this transcontinental initiative, offering nuanced insights into its environmental, economic, and socio-political dimensions.

Yang et al. (2024) examined the ecological environment, public service, and tourism economy coordination along the SREB, using a composite index system integrating single-index quantification, multi-index synthesis, and poly-criteria integration [45]. Their findings indicated spatial-temporal heterogeneity, with the Southwest region performing better than the Northwest, and revealed that tourism-related sustainability remains highly sensitive to external shocks such as political instability or pandemics. The coordinated development index was forecasted to improve, albeit with persistent regional disparities. In 2025, Li et al. focused on the dynamics of CO₂ emissions across 50 Belt and Road countries, employing longitudinal data and techniques such as spatial autocorrelation analysis and decoupling theory [46]. Their findings showed that Southeast and Central Asian nations experienced fluctuating increases in CO₂ emissions, whereas Eastern and Southern European nations showed slight declines. They recommended forming a BRI low-carbon alliance, integrating upstream-downstream industrial development, and emphasizing clean energy adoption alongside stable economic growth.

Zhu et al. (2024) analyzed the impact of BRI implementation on sustainable and inclusive development within Chinese provinces, treating the initiative as a quasi-natural experiment [47]. Their multi-dimensional index model revealed that the SREB provinces have experienced more pronounced improvements in sustainable and inclusive development compared to their Maritime Silk Road counterparts, particularly through the mechanisms of technological innovation and industrial upgrading. Jaffal (2025) provided a focused geopolitical and diplomatic analysis of China’s New Silk Road Economic Diplomacy (ED) in the Middle East, particularly through the case of the United Arab Emirates (UAE) [48]. This qualitative investigation shed light on China’s multi-pronged approach encompassing infrastructure investment, economic collaboration, cultural exchange, and regional influence. The study emphasized the strategic depth of China’s engagement and its implications for regional security and international power dynamics. From a bibliometric perspective, Youjin et al. (2024) evaluated OBOR research trends using the Web of Science Core Collection [49]. Their study revealed that environmental science and global climate change emerged as the most productive and cited domains. Although China produced the highest volume of OBOR-related research, countries with high international collaboration—such as Canada and Portugal—achieved higher citation impacts. This highlights the critical need for integrating environmental concerns in future OBOR initiatives to ensure sustainable development and global cooperation.

Together, these studies underscore the multifaceted nature of the SREB, linking environmental responsibility, diplomatic engagement, emission mitigation, regional disparity, and knowledge production as critical themes for understanding and shaping its sustainability trajectory. The current research builds upon this foundation by offering a comparative, indicator-driven sustainability assessment across SREB countries, employing an objective hybrid MCDM methodology.

While previous studies on the SREB have focused on regional coordination, CO₂ emission dynamics, and inclusive development, they often relied on descriptive models or single-country data. In contrast, this study offers a cross-national, indicator-based assessment using the MEREC-OCRA framework, enabling a structured comparison of 16 countries. Unlike qualitative or bibliometric approaches, the proposed method quantifies sustainability performance through both beneficial and non-beneficial criteria. This not only complements existing findings but also extends the discourse with a replicable, data-driven evaluation across the SREB.

## Methodology

In this study, a hybrid MCDM approach—referred to as MEREC-OCRA—is employed to assess and rank alternatives based on multiple sustainability indicators. This method integrates the MEREC for determining objective weights with the OCRA for evaluating the performance of alternatives. MEREC calculates the importance of each criterion by measuring the impact of its exclusion on the overall performance of alternatives, with higher weights assigned to those criteria whose removal results in larger deviations. The OCRA component then assesses alternatives based on both beneficial (desirable) and non-beneficial (undesirable) indicators. The final output is a comprehensive performance ranking that reflects both objective weighting and evaluation consistency. The computational steps of the MEREC-OCRA approach are outlined as follows:

Step 1: Construction of the decision matrix.

The initial decision matrix X is constructed as Eq. (1), where $x_{ij}$ denotes the performance score of alternative i with respect to criterion j, for i=1,…,n and j=1,…,m. To ensure compatibility with the MEREC method, which assumes non-negative values, a linear translation is applied if any negative values are present. This transformation retains the relative differences among values while enabling proper computational application.


$$X=[\begin{array}{cc} \begin{array}{ccc} x_{11} & x_{12} & \cdots \\ x_{21} & x_{22} & \cdots \\ \vdots & \vdots & \ddots \end{array} & \begin{array}{ccc} x_{1j} & \cdots & x_{1m}\\ x_{2j} & \cdots & x_{2m}\\ \vdots & \ddots & \vdots \end{array}\\ \begin{array}{ccc} x_{i1} & x_{i2} & \cdots \\ \vdots & \vdots & \ddots \\ x_{n1} & x_{n2} & \cdots \end{array} & \begin{array}{ccc} x_{ij} & \cdots & x_{im}\\ \vdots & \ddots & \vdots \\ x_{nj} & \cdots & x_{nm} \end{array} \end{array}]$$ (1)

Step 2: Normalization of the decision matrix.

The normalized decision matrix Y is formed to eliminate discrepancies in measurement scales, as Eq. (2). The criteria are divided into benefit-type (ℬ) and cost-type or non-benefit-type (ℋ) indicators. Normalization is performed as Eq. (3). This transformation ensures that higher values in the normalized matrix always represent more favorable outcomes.


$$Y=[\begin{array}{cc} \begin{array}{ccc} y_{11} & y_{12} & \cdots \\ y_{21} & y_{22} & \cdots \\ \vdots & \vdots & \ddots \end{array} & \begin{array}{ccc} y_{1j} & \cdots & y_{1m}\\ y_{2j} & \cdots & y_{2m}\\ \vdots & \ddots & \vdots \end{array}\\ \begin{array}{ccc} y_{i1} & y_{i2} & \cdots \\ \vdots & \vdots & \ddots \\ y_{n1} & y_{n2} & \cdots \end{array} & \begin{array}{ccc} y_{ij} & \cdots & y_{im}\\ \vdots & \ddots & \vdots \\ y_{nj} & \cdots & y_{nm} \end{array} \end{array}]$$ (2)

$$y_{ij}=\left\{ \begin{array}{cc} \frac{\min_{i}{(x}_{ij})}{x_{ij}} & \forall j\in B;i=1,\ldots ,n\;\\ \frac{x_{ij}}{\max_{i}(x_{ij})} & \forall j\in H;\;i=1,\ldots ,n \end{array}\right.\;$$ (3)

Step 3: Calculation of the initial performance score.

**Fig 1:**
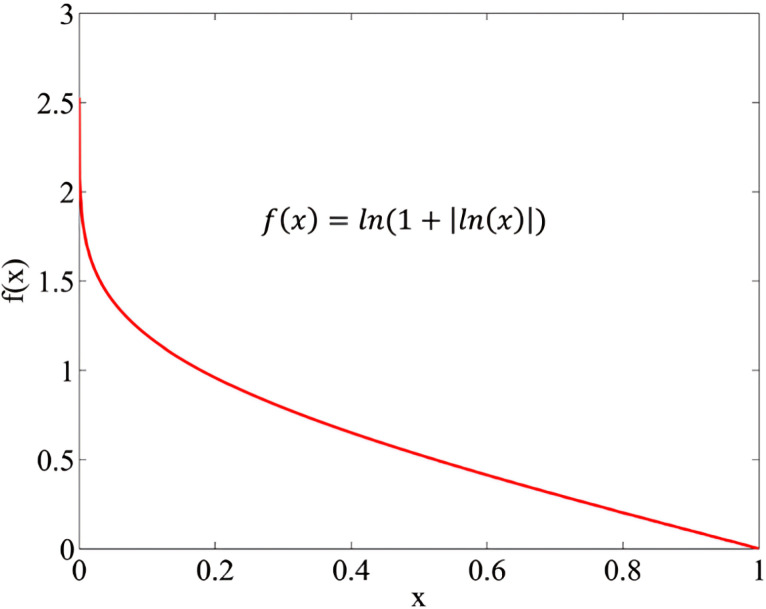
The weights of the comparative analysis.


The overall performance score $S_{i}$ of each alternative i is computed using a logarithmic aggregation function as Eq. (4) and demonstrated in Fig 1. This function balances the impact of high and low values, providing a neutral and comprehensive performance evaluation.

$$S_{i}=\ln\left(1+\left(\frac{1}{m}\sum_{j=1}^{m}\left|\ln\left(y_{ij}\right)\right|\right)\right)\;i=1,\ldots ,n$$ (4)

Step 4: Performance evaluation with eliminated criteria.

To determine the importance of each criterion, the performance score $S_{ij}^{\prime }$ is recalculated for each alternative by eliminating criterion j according to Eq. (5). These values reflect the sensitivity of each alternative’s performance to the exclusion of a specific criterion.


$$S_{ij}^{\prime }=\ln\left(1+\left(\frac{1}{m}\sum_{k=1,k\neq j}^{m}\left|\ln\left(y_{ij}\right)\right|\right)\right)\;i=1,\ldots ,n;j=1,\ldots ,m$$ (5)

Step 5: Calculation of removal effect for each criterion.

The removal effects $E_{j}$ for each criterion j is determined by the sum of absolute deviations between the original and adjusted performance scores according to Eq. (6). This value quantifies the overall influence of each criterion across all alternatives.


$$E_{j}=\sum_{i=1}^{n}\left|S_{ij}^{\prime }-S_{i}\right|\;j=1,\ldots ,m$$ (6)

Step 6: Normalization of criterion weights.

The objective weight $w_{j}$ of each criterion is calculated by normalizing the removal effects according to Eq. (7). Larger weights reflect greater influence on overall performance, ensuring that more impactful criteria are emphasized.


$$w_{j}=\frac{E_{j}}{\sum_{j=1}^{m}E_{j}}\;j=1,\ldots ,m$$ (7)

Step 7: Evaluation of non-beneficial (input) indicators.

The non-beneficial criteria are aggregated (${\overset{―}{I}}_{i}$) for each alternative i using Eq. (8).


$${\overset{―}{I}}_{i}\;=\sum_{j=1}^{g}w_{j}\left(\frac{\max_{i}\left(x_{ij}\right)-x_{ij}}{\min_{i}\left(x_{ij}\right)}\right)\;i=1,\ldots ,n$$ (8)

where *g* represents the number of non-beneficial criteria. This captures how well each alternative minimizes undesirable impacts.

Step 8: Linear transformation of non-beneficial scores

A normalized non-beneficial score $\left(\overset{―}{\overset{―}{I_{i}}}\right)$ is computed through linear scaling according to Eq. (9). This ensures that the lowest-performing alternative receives a base score of zero.


$${\overset{―}{\overset{―}{I}}}_{i}={\overset{―}{I}}_{i}-\min_{i}\left({\overset{―}{I}}_{i}\right)$$ (9)

Step 9: Evaluation of beneficial (output) indicators.

For each alternative i, the beneficial criteria are aggregated using Eq. (10). This reflects the extent to which an alternative achieves higher outcomes for favorable indicators.


$${\overset{―}{O}}_{i}=\sum_{j=g+1}^{m}w_{j}\left(\frac{x_{ij}-\min_{i}\left(x_{ij}\right)}{\min_{i}\left(x_{ij}\right)}\right)\;i=1,\ldots ,n$$ (10)

Step 10: Linear transformation of beneficial scores.

A normalized beneficial score ${\overset{―}{\overset{―}{O}}}_{i}$ is obtained using Eq. (11). This scaling ensures comparability across all alternatives.


$${\overset{―}{\overset{―}{O}}}_{i}={\overset{―}{O}}_{i}-\min_{i}\left({\overset{―}{O}}_{i}\right)$$ (11)

Step 11: Final ranking of alternatives.

The final evaluation score $P_{i}$ for each alternative is calculated by summing the normalized beneficial and non-beneficial scores as Eq. (12). Alternatives are then ranked in descending order based on $P_{i}$, with the highest score indicating the most sustainable performance.


$$P_{i}=\left({\overset{―}{\overset{―}{I}}}_{i}+{\overset{―}{\overset{―}{O}}}_{i}\right)-\min_{i}({\overset{―}{\overset{―}{I}}}_{i}+{\overset{―}{\overset{―}{O}}}_{i}\backslash \;i=1,\ldots ,n$$ (12)

### Ethical approval

This article does not contain any studies with human participants performed by any of the authors.

### Informed consent

This article does not contain any studies with human participants performed by any of the authors.

## Numerical results

In the process of selecting sustainability assessment indicators for the Silk Road Economic Belt (SREB), priority was given to a series of multidimensional indicators encompassing economic, social, and environmental aspects. The inclusion of such diverse indicators was intended to ensure that the assessment of the modern Silk Road considered not only its economic consequences but also its implications for public well-being and environmental conservation. These dimensions were selected to provide a comprehensive information base that captures the sustainability performance of countries in South Asia, West Asia, and Africa, as well as the critical challenges that must be addressed in these regions.

To ensure the credibility, transparency, and robustness of the evaluation, the data used for the 16-country sustainability assessment were collected exclusively from reputable and open-access international databases. The sources included the World Bank Open Data [50], the International Renewable Energy Agency (IRENA) [51], the International Labour Organization (ILO) [52], and the World Trade Organization (WTO) [53]. These databases are recognized for their statistical reliability and objectivity, offering standardized and peer-reviewed datasets that support valid cross-country comparisons. The most recent available data were retrieved for each indicator to ensure the findings reflect contemporary economic, environmental, and social conditions. By relying on globally trusted repositories, potential biases associated with selective reporting or outdated information were minimized, thereby strengthening the objectivity and empirical relevance of the results.

To further reinforce the real-world applicability of the proposed MEREC-OCRA model, the selected criteria were aligned with major global sustainability benchmarks, specifically the United Nations SDGs. Indicators such as greenhouse gas emissions (A12), methane emissions (A13), and nitrous oxide emissions (A14) directly reflect targets outlined in SDG 13 (Climate Action). Similarly, GDP per capita (A2), human capital index (A10), and gross capital formation (A11) correspond to SDGs 8 (Decent Work and Economic Growth) and 9 (Industry, Innovation, and Infrastructure). The alignment of the chosen indicators with internationally recognized goals validates the MEREC-OCRA framework as an appropriate and meaningful tool for assessing sustainability in complex, real-world contexts. Although empirical case studies were not conducted, the model’s outcomes show strong coherence with well-established sustainability trends and global development priorities, thereby affirming the external validity of the proposed methodological approach.

A total of 14 sustainability indicators were selected and categorized into beneficial and non-beneficial criteria, as detailed in Table 2. These indicators served as the foundation for building the decision matrix. Each column in the matrix corresponds to a specific criterion, while each row represents one of the 16 countries under consideration. The MEREC-OCRA method, described in detail in the Methodology section, was then applied to evaluate the sustainability performance of the countries. This was done by first constructing a decision matrix (Table 3), followed by a normalization process (Table 4) using Equation (3) to convert all values into a common scale that accommodates both beneficial and non-beneficial criteria. This transformation enabled a consistent and fair comparison of all countries across all selected dimensions.

**Table 2:** The description of considered criteria.

No.	Criteria	Notation
1	Agriculture, forestry, and fishing, value added (% of GDP)	A1
2	GDP per capita (current thousand US$)	A2
3	Gross national expenditure (% of GDP)	A3
4	Inflation, consumer prices (annual %)	A4
5	Inflation, GDP deflator (annual %)	A5
6	Exports of goods and services (% of GDP)	A6
7	Imports of goods and services (% of GDP)	A7
8	Labor force, total (million people)	A8
9	Armed forces personnel (% of total labor force)	A9
10	Human capital index (HCI) (scale 0–1)	A10
11	Gross capital formation (% of GDP)	A11
12	Total greenhouse gas emissions (Mt of CO2 equivalent)	A12
13	Methane emissions in energy sector (Million metric tons of CO2 equivalent)	A13
14	Nitrous oxide emissions in energy sector (Million metric tons of CO2 equivalent)	A14

**Table 3:** The decision matrix.

Country	Criteria													
	A1	A2	A3	A4	A5	A6	A7	A8	A9	A10	A11	A12	A13	A14
Iraq	2.85	5.94	78.20	4.99	18.87	37.68	24.41	1.11	4.30	0.41	19.59	261.29	70.02	0.73
Turkey	6.46	10.62	104.71	72.31	96.11	37.89	42.60	34.43	1.62	0.65	35.05	504.96	9.93	2.11
Lebanon	1.40	4.14	126.55	171.21	150.00	26.03	52.80	1.87	4.39	0.52	5.36	28.95	0.12	0.18
Israel	1.26	54.66	96.71	4.39	4.39	31.91	28.62	4.37	4.36	0.73	26.27	83.66	0.79	0.45
Saudi Arabia	2.41	30.44	83.30	2.47	17.32	39.92	23.22	15.91	1.76	0.58	27.28	712.59	70.87	2.91
Oman	1.84	25.06	88.86	2.81	24.65	52.53	41.39	2.24	2.13	0.61	22.41	95.08	17.65	0.26
UAE	0.91	53.76	77.41	4.83	13.85	95.93	70.65	6.58	0.97	0.67	22.79	249.93	33.77	0.78
Qatar	0.25	88.05	75.22	5.00	25.99	58.91	34.13	2.00	1.07	0.64	36.68	119.61	28.91	0.33
Kuwait	0.46	43.23	91.60	3.98	26.62	53.29	44.89	2.42	1.04	0.56	25.02	135.90	20.09	0.36
Bahrain	0.26	30.15	80.52	3.63	7.71	89.65	70.17	0.84	2.29	0.65	25.58	54.15	4.66	0.09
Egypt	10.95	4.30	106.81	13.90	10.43	15.09	21.90	31.17	2.80	0.49	17.02	299.78	25.36	1.14
India	16.62	2.39	102.19	6.70	8.32	22.45	26.92	52.38	0.62	0.49	31.16	3200.82	124.66	21.58
Pakistan	22.25	1.60	111.38	19.87	13.00	10.47	21.85	78.91	1.29	0.41	15.14	436.61	23.98	3.11
Bangladesh	11.22	2.69	106.82	7.70	5.05	12.88	20.90	74.46	0.32	0.46	32.05	206.57	8.86	7.64
Sri Lanka	8.75	3.35	103.55	49.72	48.85	21.48	25.04	8.71	3.68	0.60	34.39	35.12	0.75	0.43
Nepal	21.06	1.34	131.64	7.69	7.33	6.76	42.64	8.70	1.37	0.50	37.42	45.87	3.16	0.61

**Table 4:** The normalized decision matrix.

Country	Criteria													
	A1	A2	A3	A4	A5	A6	A7	A8	A9	A10	A11	A12	A13	A14
Iraq	0.089	0.225	0.962	0.029	0.126	0.179	0.856	0.075	0.074	0.996	0.274	0.111	0.562	0.034
Turkey	0.039	0.126	0.718	0.422	0.641	0.178	0.491	0.024	0.198	0.625	0.153	0.057	0.080	0.098
Lebanon	0.182	0.323	0.594	1.000	1.000	0.260	0.396	0.451	0.073	0.788	1.000	1.000	0.001	0.008
Israel	0.202	0.024	0.778	0.026	0.029	0.212	0.730	0.193	0.073	0.553	0.204	0.346	0.006	0.021
Saudi Arabia	0.106	0.044	0.903	0.014	0.115	0.169	0.900	0.053	0.181	0.705	0.196	0.041	0.569	0.135
Oman	0.138	0.053	0.846	0.016	0.164	0.129	0.505	0.375	0.150	0.668	0.239	0.304	0.142	0.012
UAE	0.279	0.025	0.972	0.028	0.092	0.070	0.296	0.128	0.330	0.603	0.235	0.116	0.271	0.036
Qatar	1.000	0.015	1.000	0.029	0.173	0.115	0.612	0.420	0.298	0.637	0.146	0.242	0.232	0.015
Kuwait	0.557	0.031	0.821	0.023	0.177	0.127	0.466	0.348	0.309	0.722	0.214	0.213	0.161	0.017
Bahrain	0.989	0.044	0.934	0.021	0.051	0.075	0.298	1.000	0.140	0.622	0.210	0.535	0.037	0.004
Egypt	0.023	0.311	0.704	0.081	0.070	0.448	0.954	0.027	0.114	0.821	0.315	0.097	0.203	0.053
India	0.015	0.560	0.736	0.039	0.055	0.301	0.776	0.002	0.518	0.823	0.172	0.009	1.000	1.000
Pakistan	0.011	0.837	0.675	0.116	0.087	0.646	0.956	0.011	0.247	1.000	0.354	0.066	0.192	0.144
Bangladesh	0.023	0.497	0.704	0.045	0.034	0.525	1.000	0.011	1.000	0.875	0.167	0.140	0.071	0.035
Sri Lanka	0.029	0.398	0.726	0.290	0.326	0.315	0.835	0.097	0.087	0.679	0.156	0.824	0.006	0.020
Nepal	0.012	1.000	0.571	0.045	0.049	1.000	0.490	0.097	0.233	0.805	0.143	0.631	0.025	0.028

**Fig 2:**
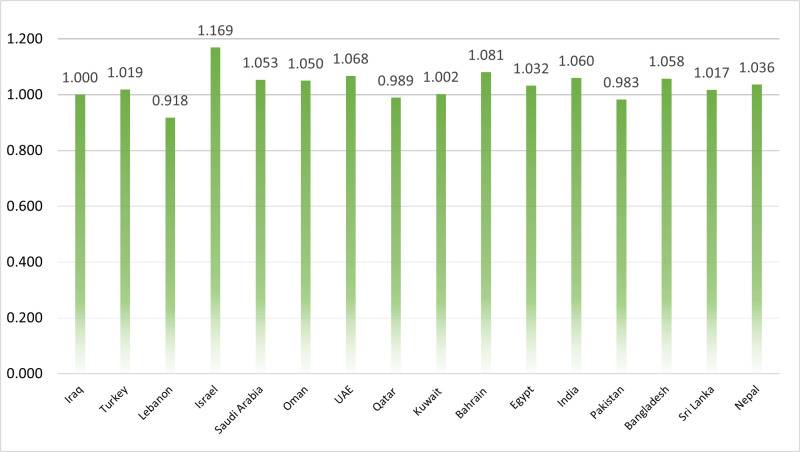
The overall performance of studied countries.

At this essential stage of the decision-making process, a comprehensive evaluation of the overall performance of all available alternatives is considered imperative. This critical assessment is carried out through the implementation of Eq. (4), which functions as the primary computational tool for quantifying the consolidated performance score of each alternative. Through the application of this equation, a numerical representation of each alternative’s relative efficacy and suitability—based on the full set of evaluation criteria—is obtained. The results derived from these calculations are systematically illustrated in Fig 2, which presents the total performance scores of all countries under consideration. As depicted in Fig 2, the performance levels across the assessed countries are relatively comparable. The highest total performance score is observed for Israel, which achieved a value of 1.169, whereas the lowest score is recorded for Lebanon, with a value of 0.918. The narrow range of these scores indicates that the countries exhibit limited variance in overall sustainability performance.

**Fig 3:**
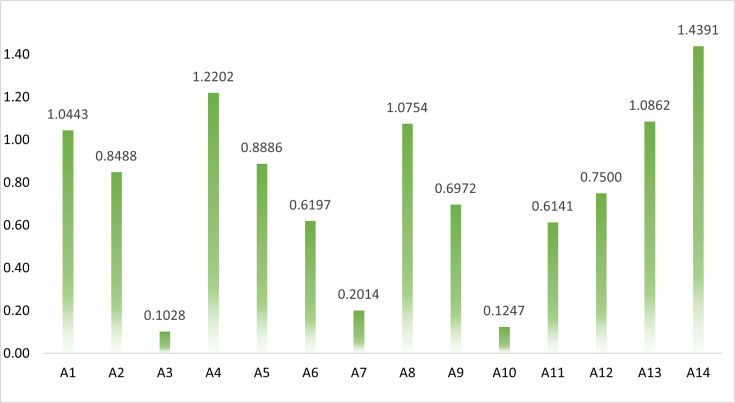
The efficiency of eliminating criterion.

**Fig 4:**
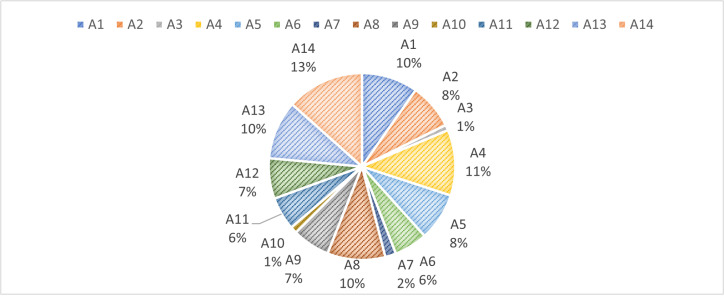
The obtained criteria weight.

Upon the completion of this initial evaluation, the decision-making framework progresses to the next stage, which involves a more granular analysis of the criteria’s individual contributions. At this point, the removal of each criterion is simulated to assess its influence on the alternatives’ total performance. This is conducted using Eq. (5), which recalculates the performance of each alternative in the absence of a specific criterion $S_{ij}^{\prime }$. The resulting performance deviations are compiled and displayed in Table 5, offering a detailed account of how each criterion affects the evaluation outcomes. Subsequently, the removal effects of all criteria are quantified by employing Eq. (6). These values, which reflect the aggregated impact of criterion exclusion across all alternatives, are visualized in Fig 3. Finally, the objective weights of the criteria are derived by normalizing the removal effect values through the use of Eq. (7). The outcome of this step, as illustrated in Fig 4, provides a transparent and data-driven determination of the relative importance of each evaluation criterion in shaping the sustainability rankings.

**Table 5:** The eliminated overall performance.

Country	Criteria													
	A1	A2	A3	A4	A5	A6	A7	A8	A9	A10	A11	A12	A13	A14
Iraq	0.935	0.960	0.999	0.903	0.944	0.954	0.996	0.930	0.930	1.000	0.966	0.941	0.985	0.907
Turkey	0.932	0.964	1.010	0.996	1.007	0.973	1.000	0.918	0.976	1.006	0.969	0.942	0.951	0.957
Lebanon	0.868	0.885	0.903	0.918	0.918	0.879	0.891	0.895	0.840	0.911	0.918	0.918	0.696	0.771
Israel	1.133	1.083	1.163	1.084	1.087	1.134	1.162	1.132	1.109	1.156	1.133	1.145	1.050	1.079
Saudi Arabia	0.996	0.972	1.051	0.942	0.998	1.008	1.051	0.977	1.010	1.045	1.012	0.970	1.039	1.002
Oman	0.999	0.974	1.046	0.942	1.004	0.998	1.033	1.025	1.002	1.040	1.014	1.020	1.000	0.933
UAE	1.036	0.972	1.067	0.976	1.007	1.000	1.037	1.016	1.040	1.055	1.031	1.013	1.035	0.983
Qatar	0.989	0.871	0.989	0.890	0.941	0.930	0.976	0.966	0.956	0.977	0.937	0.951	0.949	0.871
Kuwait	0.987	0.907	0.997	0.898	0.956	0.947	0.982	0.974	0.971	0.994	0.961	0.961	0.953	0.889
Bahrain	1.081	1.002	1.079	0.983	1.006	1.016	1.051	1.081	1.032	1.069	1.042	1.066	0.998	0.937
Egypt	0.932	1.002	1.023	0.966	0.962	1.012	1.031	0.936	0.976	1.027	1.002	0.971	0.991	0.955
India	0.951	1.045	1.052	0.976	0.986	1.030	1.054	0.886	1.043	1.055	1.015	0.936	1.060	1.060
Pakistan	0.855	0.978	0.972	0.923	0.915	0.971	0.981	0.853	0.944	0.983	0.954	0.907	0.937	0.929
Bangladesh	0.959	1.040	1.049	0.978	0.970	1.042	1.058	0.940	1.058	1.055	1.013	1.008	0.990	0.971
Sri Lanka	0.922	0.993	1.009	0.985	0.988	0.987	1.013	0.955	0.952	1.007	0.968	1.012	0.876	0.911
Nepal	0.917	1.036	1.022	0.954	0.957	1.036	1.018	0.975	0.999	1.031	0.986	1.024	0.938	0.942

In the light of Fig 4, it is observed that the most influential criterion is A14 – Nitrous oxide emissions in the energy sector, which received the highest weight of 0.1343. This indicates that variations in nitrous oxide emissions play a significant role in shaping the overall sustainability rankings. The prominence of this environmental indicator underscores the growing concern over energy-related emissions and their ecological impact, especially in regions facing intensive industrial activity and weak environmental regulations. Similarly, A4 – Inflation (consumer prices) and A13 – Methane emissions in the energy sector also received high weights of 0.1139 and 0.1014, respectively. These criteria reflect both macroeconomic stability and climate-related pressures, suggesting that sustainable development in the studied regions is highly sensitive to both environmental emissions and economic volatility. Inflation, as a key economic indicator, influences purchasing power and investment attractiveness, while methane emissions highlight the broader environmental footprint of the energy sector. The criterion A8 – Labor force, total (million people) was also assigned a substantial weight of 0.1004, indicating the strong role that human capital and labor capacity play in determining development potential. A large labor force, if effectively mobilized, may support sustainable economic expansion, provided that employment is aligned with green and inclusive growth strategies.

In contrast, several criteria were found to exert relatively lower influence. Notably, A3 – Gross national expenditure (% of GDP) and A10 – Human Capital Index (HCI) received weights of 0.0096 and 0.0116, respectively. The low weight of gross national expenditure may suggest that this indicator varies less across the countries or has a limited direct correlation with broader sustainability outcomes in the SREB context. Similarly, the human capital index—despite its conceptual relevance—may display minimal variability or overlapping values in the studied dataset, thus reducing its discrimination power in the decision model. Moderate weights were observed for other economic and environmental indicators, such as A1 – Agriculture, forestry, and fishing (0.0975), A2 – GDP per capita (0.0792), A5 – GDP deflator (0.0830), and A12 – Greenhouse gas emissions (0.0700). These indicators contribute meaningfully to the evaluation but are not as dominant as A14 or A4. Their inclusion highlights the multidimensional nature of sustainability, where both productive capacity and ecological stability interact to shape national development trajectories.

The aggregation of the performance scores for all non-beneficial criteria across the evaluated alternatives has been conducted through the application of Eq. (8) as shown in Table 6. This procedure enables the construction of a composite matrix reflecting the cumulative negative impacts associated with each alternative. To evaluate the overall performance of the alternatives with respect to beneficial criteria, Eq. (10) as shown in Table 7.

**Table 6:** The weighted aggregated performance scores for non-beneficial criteria.

Country	Non-benefit criteria				${\overset{―}{I}}_{i}$
	A4	A5	A13	A14	
Iraq	7.652	2.477	47.926	32.756	90.811
Turkey	4.553	1.018	100.637	30.592	136.800
Lebanon	0	0	109.244	33.629	142.872
Israel	7.680	2.751	108.654	33.208	152.292
Saudi Arabia	7.768	2.506	47.180	29.334	86.789
Oman	7.752	2.368	93.863	33.508	137.491
UAE	7.660	2.572	79.728	32.679	122.639
Qatar	7.652	2.343	83.983	33.390	127.368
Kuwait	7.699	2.331	91.727	33.346	135.102
Bahrain	7.715	2.688	105.262	33.776	149.441
Egypt	7.242	2.637	87.103	32.114	129.095
India	7.574	2.676	0	0	10.250
Pakistan	6.967	2.588	88.309	29.024	126.888
Bangladesh	7.528	2.738	101.577	32.710	144.553
Sri Lanka	5.593	1.911	108.686	33.240	149.430
Nepal	7.528	2.695	106.576	32.946	149.745

**Table 7:** The weighted aggregated performance scores for beneficial criteria.

Country	Beneficial Criteria										${\overset{―}{O}}_{i}$
	A1	A2	A3	A6	A7	A8	A9	A10	A11	A12	
Iraq	0.9932	0.2727	0.0004	0.2646	0.0032	1.2325	0.8105	0.0001	0.1522	0.5619	80.561
Turkey	2.3737	0.5501	0.0038	0.2664	0.0195	4.0028	0.2640	0.0070	0.3176	1.1512	126.551
Lebanon	0.4381	0.1660	0.0066	0.1648	0.0287	0.1221	0.8279	0.0031	0	0	132.623
Israel	0.3849	3.1612	0.0027	0.2152	0.0070	0.4205	0.8210	0.0094	0.2237	0.1323	142.042
Saudi Arabia	0.8228	1.7251	0.0010	0.2837	0.0021	1.7962	0.2935	0.0049	0.2344	1.6534	76.539
Oman	0.6066	1.4062	0.0017	0.3916	0.0184	0.1670	0.3687	0.0058	0.1824	0.1599	127.241
UAE	0.2521	3.1077	0.0003	0.7629	0.0448	0.6837	0.1323	0.0077	0.1864	0.5344	112.389
Qatar	0	5.1405	0	0.4462	0.0119	0.1384	0.1535	0.0066	0.3350	0.2193	117.118
Kuwait	0.0775	2.4838	0.0021	0.3981	0.0216	0.1880	0.1458	0.0045	0.2103	0.2587	124.852
Bahrain	0.0011	1.7083	0.0007	0.7092	0.0443	0	0.3998	0.0071	0.2162	0.0610	139.191
Egypt	4.0893	0.1754	0.0040	0.0712	0.0009	3.6141	0.5052	0.0025	0.1247	0.6550	118.845
India	6.2591	0.0624	0.0034	0.1342	0.0054	62.3318	0.0605	0.0025	0.2759	7.6711	0
Pakistan	8.4135	0.0154	0.0046	0.0317	0.0009	9.3042	0.1982	0	0.1046	0.9859	116.638
Bangladesh	4.1933	0.0801	0.0040	0.0524	0	8.7738	0	0.0017	0.2854	0.4296	134.303
Sri Lanka	3.2491	0.1196	0.0036	0.1260	0.0037	0.9382	0.6829	0.0055	0.3105	0.0149	139.180
Nepal	7.9584	0	0.0072	0	0.0196	0.9366	0.2139	0.0028	0.3429	0.0409	139.495

**Fig 5:**
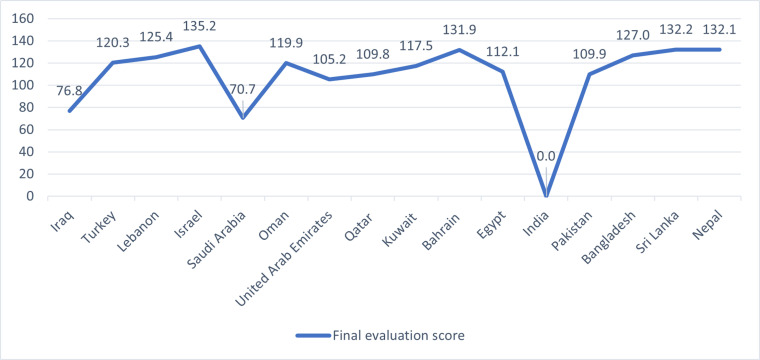
The final evaluation score of studied countries in SREB.

Following this step, the linear priority ranking of the alternatives, based solely on non-beneficial and beneficial criteria indicators, is determined using Eq. (9) and Eq. (11), which standardizes and scales the resulting values for comparative purposes. The computed results derived from both non-beneficial and beneficial evaluations are presented in Table 8. A synthesized visualization of these outcomes, highlighting the final evaluation score $P_{i}$ of all alternatives according to Eq. (12), is illustrated in Fig 5. The final evaluation scores, as obtained from the application of the MEREC-OCRA method, provide a comprehensive ranking of the sixteen countries assessed under the SREB framework. These scores reflect the integrated sustainability performance of each country, incorporating both beneficial and non-beneficial criteria related to environmental, social, and economic dimensions. Among all participating countries, Israel achieved the highest overall score (135.22), indicating its leading position in terms of sustainability performance. This result suggests a strong balance between economic productivity, environmental responsibility, and social development. Close behind Israel are Sri Lanka (132.18), Bahrain (131.86), and Nepal (132.09), all of which demonstrate commendable performance across the assessed criteria. These high-ranking countries may be characterized by effective emission management, robust human capital indicators, or favorable macroeconomic conditions. Bangladesh (127.01), Lebanon (125.44), and Turkey (120.28) also occupy relatively strong positions within the ranking. Their mid-to-upper-tier placement reflects positive achievements in specific areas, such as workforce size, export orientation, or controlled inflation, although further improvements may be necessary in environmental or capital formation indicators.

**Table 8:** The Linear transformation of non-beneficial and beneficial scores.

Country	${\overset{―}{\overset{―}{I}}}_{i}$	${\overset{―}{\overset{―}{O}}}_{i}$
Iraq	80.561	3.691
Turkey	126.551	1.139
Lebanon	132.623	0.230
Israel	142.042	0.586
Saudi Arabia	76.539	1.586
Oman	127.241	0.116
UAE	112.389	0.260
Qatar	117.118	0.114
Kuwait	124.852	0.019
Bahrain	139.191	0.083
Egypt	118.845	0.687
India	0	7.409
Pakistan	116.638	0.688
Bangladesh	134.303	0.116
Sri Lanka	139.180	0.413
Nepal	139.495	0

On the other hand, India received the lowest evaluation score (0), signifying considerable challenges in achieving sustainable development objectives as measured by the selected indicators. This may be attributed to high emissions, volatility in macroeconomic indicators such as inflation, or insufficient gains in capital formation and per capita productivity. Saudi Arabia (70.71) and Iraq (76.84) also rank near the bottom, which may reflect ongoing structural vulnerabilities, dependence on resource extraction, or environmental externalities. In the middle range, countries such as Oman (119.95), Kuwait (117.46), Egypt (112.12), Pakistan (109.92), and Qatar (109.82) exhibit relatively balanced performance but with notable room for enhancement. While some of these nations demonstrate progress in economic indicators, environmental sustainability—particularly related to greenhouse gas emissions and energy sector pollutants—may remain a critical constraint. The results also highlight the nuanced performance of United Arab Emirates (105.24), which, despite being a high-income country, may face sustainability trade-offs related to its carbon-intensive economic structure and urban growth patterns.

## Sensitivity analysis

To evaluate the robustness and stability of the country rankings produced by the MEREC-OCRA method, a sensitivity analysis was performed by applying the same objective MEREC-derived weights to two additional MCDM techniques: TOPSIS (Technique for Order of Preference by Similarity to Ideal Solution) and EDAS (Evaluation based on Distance from Average Solution). These methods were selected for their compatibility with objective weighting and their ability to differentiate among alternatives using both beneficial and non-beneficial criteria.

**Fig 6:**
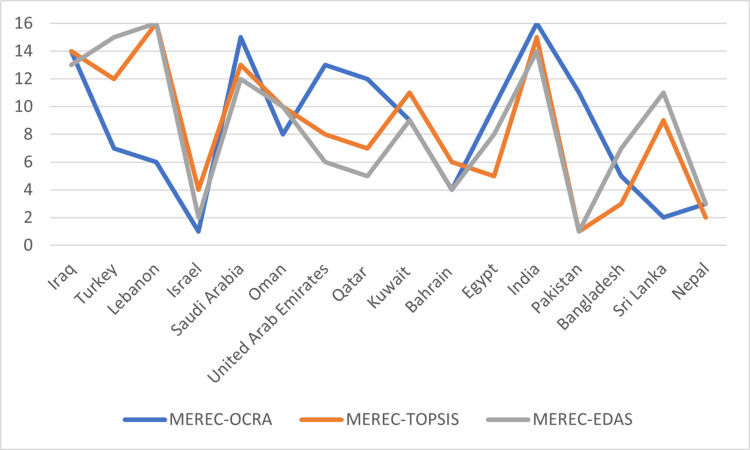
The sensitivity analysis results.

The comparison results of the country rankings derived from the three methods are presented visually depicted in Fig 6. The rankings demonstrate a high degree of consistency in identifying countries with the best and worst performance in terms of sustainability. Notably, Israel, Nepal, and Pakistan consistently appear among the top-performing countries, while India, Saudi Arabia, and Iraq are ranked toward the bottom across all three methods. Minor discrepancies in middle-ranking countries—such as Lebanon, Sri Lanka, and Turkey—can be attributed to differences in how each method handles normalization and distance computations. Fig 6 illustrates the comparative rankings across all methods, highlighting both convergence and divergence. The overall similarity of the results supports the robustness of the MEREC-OCRA model and its capacity to deliver stable, reproducible decisions even when cross-validated with alternative MCDM approaches.

## Policy recommendations

Based on the final evaluation results derived from the MEREC-OCRA method, a series of actionable recommendations are proposed to support policymakers across South Asia, West Asia, and Africa in enhancing sustainability outcomes within the SREB context. These recommendations are informed by the weighted criteria and observed performance disparities among the sixteen evaluated countries.

First, it is recommended that countries exhibiting lower sustainability scores—such as India, Iraq, and Saudi Arabia—prioritize the development and enforcement of comprehensive climate and emissions policies. Particular attention should be paid to reducing methane and nitrous oxide emissions, which were identified as the most influential indicators in the assessment. Investments should be directed toward low-carbon technologies and infrastructure upgrades in energy-intensive sectors. Additionally, the introduction of emissions trading systems, renewable energy incentives, and stricter environmental compliance mechanisms would contribute to reducing harmful outputs and aligning national goals with the global climate agenda. Macroeconomic stability also emerged as a critical factor, with inflation-related indicators carrying considerable weight. Countries facing high levels of economic volatility are encouraged to implement fiscal and monetary policy reforms that promote price stability and protect vulnerable populations. This may include the adoption of inflation-targeting regimes, improved central bank independence, and the expansion of price stabilization funds and social protection schemes to mitigate economic shocks.

Human capital development represents another priority area, particularly for nations with large labor forces but relatively weak educational or health infrastructure. In countries such as India and Pakistan, national strategies should focus on enhancing labor productivity through increased investment in education, vocational training, and technical skills development. Particular emphasis should be placed on aligning workforce development with emerging green industries to ensure that employment growth supports both social and environmental objectives. Furthermore, agricultural sustainability and land-use efficiency should be strengthened, especially in economies where agriculture remains a key economic driver. The adoption of climate-smart agriculture, sustainable irrigation practices, and integrated land management systems is strongly recommended. Support programs for smallholder farmers, including training, cooperative models, and financial inclusion mechanisms, would also be beneficial in improving rural livelihoods and ecosystem resilience. It is also recommended that high-performing countries—such as Israel, Sri Lanka, and Nepal—be positioned as regional role models for sustainability planning and implementation. Platforms for cross-border policy dialogue and knowledge-sharing should be institutionalized under the SREB framework, allowing for best practices and innovations to be disseminated. These collaborations could include joint environmental monitoring initiatives, regional benchmarking tools, and technical assistance programs to harmonize sustainability efforts.

Lastly, the institutionalization of data-driven decision-making should be pursued across all participating countries. The establishment of national sustainability observatories, improvements in statistical capacity, and the deployment of open-data platforms are essential for monitoring progress and guiding policy adaptations. Transparent, timely, and reliable data will empower policymakers to track developments, identify gaps, and respond effectively to dynamic sustainability challenges.

## Conclusion

This study was embedded within the expansive and historic context of the SREB, which is designed to rekindle ancient trade routes across Asia, Europe, and Africa. The research was motivated by the urgent need to evaluate sustainable development across this vast network, particularly in the dynamically evolving regions of South Asia, West Asia, and Africa. These regions are characterized by a rich mosaic of geographical, cultural, and socio-economic diversity, with each area facing distinctive challenges in achieving sustainable development. The study was undertaken with the aim of addressing the gap in comprehensive assessments of the environmental and economic impacts of the SREB, thereby providing an evidence-based foundation to inform and guide future policy and development strategies.

A novel methodological framework, known as the MEREC-OCRA method, was introduced and employed in this study. This approach integrates the objective weighting capabilities of the MEREC method—which assesses the impact of criteria removal on overall alternative performance—with the operational competitiveness evaluation offered by the OCRA technique. By applying this hybrid multi-criteria decision-making framework, an objective and reproducible ranking of sixteen countries involved in the SREB was achieved. The methodological innovation was designed to address both beneficial and non-beneficial sustainability indicators, thereby providing a comprehensive and balanced assessment of each country’s performance.

The application of the MEREC-OCRA methodology yielded a spectrum of sustainability performance scores, which revealed significant disparities among the countries examined. Some nations were identified as leaders in sustainable development, while others were found to lag considerably behind. The results provided insightful benchmarks that illustrated the varying effectiveness of sustainable practices and policies across the evaluated regions. Through these findings, a foundational understanding was established regarding the relative progress of each country and the overall challenges they face in navigating their sustainable development trajectories.

While the study yielded groundbreaking insights, it was recognized that inherent limitations exist, particularly due to the reliance on decision-makers’ judgments that may introduce subjective biases. As a consequence, it is proposed that future research should apply the MEREC-OCRA method to a broader range of multi-criteria decision-making problems across different geographical and situational contexts. Such expansion could involve diverse comparative analyses employing various sets of criteria and alternatives, thereby enhancing the robustness and generalizability of the findings. Further research is also recommended to validate the current results against empirical case studies and establish sustainability benchmarks, such as those defined by the United Nations Sustainable Development Goals, in order to further enrich the discourse on sustainable development evaluation.

## Supporting information

S1 File The decision making data and computation results. (DOCX)
