# Supplementary material for: Assessing sustainability on the modern Silk Road: An objective weighting methodological approach
Source: PLoS One. 2025 May 20;20(5):e0324538. doi: 10.1371/journal.pone.0324538 (PMC12091899; doi:10.1371/journal.pone.0324538)
Supplement: S1 File — (DOCX) [file pone.0324538.s001.docx]

**Minimal Data Set**

1. Decision matrix

| Country | Criteria | | | | | | | | | | | | | |
| --- | --- | --- | --- | --- | --- | --- | --- | --- | --- | --- | --- | --- | --- | --- |
|  | A1 | A2 | A3 | A4 | A5 | A6 | A7 | A8 | A9 | A10 | A11 | A12 | A13 | A14 |
| Iraq | 2.85 | 5.94 | 78.20 | 4.99 | 18.87 | 37.68 | 24.41 | 1.11 | 4.30 | 0.41 | 19.59 | 261.29 | 70.02 | 0.73 |
| Turkey | 6.46 | 10.62 | 104.71 | 72.31 | 96.11 | 37.89 | 42.60 | 34.43 | 1.62 | 0.65 | 35.05 | 504.96 | 9.93 | 2.11 |
| Lebanon | 1.40 | 4.14 | 126.55 | 171.21 | 150.00 | 26.03 | 52.80 | 1.87 | 4.39 | 0.52 | 5.36 | 28.95 | 0.12 | 0.18 |
| Israel | 1.26 | 54.66 | 96.71 | 4.39 | 4.39 | 31.91 | 28.62 | 4.37 | 4.36 | 0.73 | 26.27 | 83.66 | 0.79 | 0.45 |
| Saudi Arabia | 2.41 | 30.44 | 83.30 | 2.47 | 17.32 | 39.92 | 23.22 | 15.91 | 1.76 | 0.58 | 27.28 | 712.59 | 70.87 | 2.91 |
| Oman | 1.84 | 25.06 | 88.86 | 2.81 | 24.65 | 52.53 | 41.39 | 2.24 | 2.13 | 0.61 | 22.41 | 95.08 | 17.65 | 0.26 |
| UAE | 0.91 | 53.76 | 77.41 | 4.83 | 13.85 | 95.93 | 70.65 | 6.58 | 0.97 | 0.67 | 22.79 | 249.93 | 33.77 | 0.78 |
| Qatar | 0.25 | 88.05 | 75.22 | 5.00 | 25.99 | 58.91 | 34.13 | 2.00 | 1.07 | 0.64 | 36.68 | 119.61 | 28.91 | 0.33 |
| Kuwait | 0.46 | 43.23 | 91.60 | 3.98 | 26.62 | 53.29 | 44.89 | 2.42 | 1.04 | 0.56 | 25.02 | 135.90 | 20.09 | 0.36 |
| Bahrain | 0.26 | 30.15 | 80.52 | 3.63 | 7.71 | 89.65 | 70.17 | 0.84 | 2.29 | 0.65 | 25.58 | 54.15 | 4.66 | 0.09 |
| Egypt | 10.95 | 4.30 | 106.81 | 13.90 | 10.43 | 15.09 | 21.90 | 31.17 | 2.80 | 0.49 | 17.02 | 299.78 | 25.36 | 1.14 |
| India | 16.62 | 2.39 | 102.19 | 6.70 | 8.32 | 22.45 | 26.92 | 52.38 | 0.62 | 0.49 | 31.16 | 3200.82 | 124.66 | 21.58 |
| Pakistan | 22.25 | 1.60 | 111.38 | 19.87 | 13.00 | 10.47 | 21.85 | 78.91 | 1.29 | 0.41 | 15.14 | 436.61 | 23.98 | 3.11 |
| Bangladesh | 11.22 | 2.69 | 106.82 | 7.70 | 5.05 | 12.88 | 20.90 | 74.46 | 0.32 | 0.46 | 32.05 | 206.57 | 8.86 | 7.64 |
| Sri Lanka | 8.75 | 3.35 | 103.55 | 49.72 | 48.85 | 21.48 | 25.04 | 8.71 | 3.68 | 0.60 | 34.39 | 35.12 | 0.75 | 0.43 |
| Nepal | 21.06 | 1.34 | 131.64 | 7.69 | 7.33 | 6.76 | 42.64 | 8.70 | 1.37 | 0.50 | 37.42 | 45.87 | 3.16 | 0.61 |

1. Normalized decision matrix

| Country | Criteria | | | | | | | | | | | | | |
| --- | --- | --- | --- | --- | --- | --- | --- | --- | --- | --- | --- | --- | --- | --- |
|  | A1 | A2 | A3 | A4 | A5 | A6 | A7 | A8 | A9 | A10 | A11 | A12 | A13 | A14 |
| Iraq | 0.089 | 0.225 | 0.962 | 0.029 | 0.126 | 0.179 | 0.856 | 0.075 | 0.074 | 0.996 | 0.274 | 0.111 | 0.562 | 0.034 |
| Turkey | 0.039 | 0.126 | 0.718 | 0.422 | 0.641 | 0.178 | 0.491 | 0.024 | 0.198 | 0.625 | 0.153 | 0.057 | 0.080 | 0.098 |
| Lebanon | 0.182 | 0.323 | 0.594 | 1.000 | 1.000 | 0.260 | 0.396 | 0.451 | 0.073 | 0.788 | 1.000 | 1.000 | 0.001 | 0.008 |
| Israel | 0.202 | 0.024 | 0.778 | 0.026 | 0.029 | 0.212 | 0.730 | 0.193 | 0.073 | 0.553 | 0.204 | 0.346 | 0.006 | 0.021 |
| Saudi Arabia | 0.106 | 0.044 | 0.903 | 0.014 | 0.115 | 0.169 | 0.900 | 0.053 | 0.181 | 0.705 | 0.196 | 0.041 | 0.569 | 0.135 |
| Oman | 0.138 | 0.053 | 0.846 | 0.016 | 0.164 | 0.129 | 0.505 | 0.375 | 0.150 | 0.668 | 0.239 | 0.304 | 0.142 | 0.012 |
| UAE | 0.279 | 0.025 | 0.972 | 0.028 | 0.092 | 0.070 | 0.296 | 0.128 | 0.330 | 0.603 | 0.235 | 0.116 | 0.271 | 0.036 |
| Qatar | 1.000 | 0.015 | 1.000 | 0.029 | 0.173 | 0.115 | 0.612 | 0.420 | 0.298 | 0.637 | 0.146 | 0.242 | 0.232 | 0.015 |
| Kuwait | 0.557 | 0.031 | 0.821 | 0.023 | 0.177 | 0.127 | 0.466 | 0.348 | 0.309 | 0.722 | 0.214 | 0.213 | 0.161 | 0.017 |
| Bahrain | 0.989 | 0.044 | 0.934 | 0.021 | 0.051 | 0.075 | 0.298 | 1.000 | 0.140 | 0.622 | 0.210 | 0.535 | 0.037 | 0.004 |
| Egypt | 0.023 | 0.311 | 0.704 | 0.081 | 0.070 | 0.448 | 0.954 | 0.027 | 0.114 | 0.821 | 0.315 | 0.097 | 0.203 | 0.053 |
| India | 0.015 | 0.560 | 0.736 | 0.039 | 0.055 | 0.301 | 0.776 | 0.002 | 0.518 | 0.823 | 0.172 | 0.009 | 1.000 | 1.000 |
| Pakistan | 0.011 | 0.837 | 0.675 | 0.116 | 0.087 | 0.646 | 0.956 | 0.011 | 0.247 | 1.000 | 0.354 | 0.066 | 0.192 | 0.144 |
| Bangladesh | 0.023 | 0.497 | 0.704 | 0.045 | 0.034 | 0.525 | 1.000 | 0.011 | 1.000 | 0.875 | 0.167 | 0.140 | 0.071 | 0.035 |
| Sri Lanka | 0.029 | 0.398 | 0.726 | 0.290 | 0.326 | 0.315 | 0.835 | 0.097 | 0.087 | 0.679 | 0.156 | 0.824 | 0.006 | 0.020 |
| Nepal | 0.012 | 1.000 | 0.571 | 0.045 | 0.049 | 1.000 | 0.490 | 0.097 | 0.233 | 0.805 | 0.143 | 0.631 | 0.025 | 0.028 |

1. The overall performance of studied countries

| Country | $S_{i}$ |
| --- | --- |
| Iraq | 1.00032436 |
| Turkey | 1.01861374 |
| Lebanon | 0.91783697 |
| Israel | 1.16897921 |
| Saudi Arabia | 1.05338614 |
| Oman | 1.05013662 |
| United Arab Emirates | 1.06756996 |
| Qatar | 0.98901324 |
| Kuwait | 1.00228043 |
| Bahrain | 1.0808881 |
| Egypt | 1.03233576 |
| India | 1.05982848 |
| Pakistan | 0.98251462 |
| Bangladesh | 1.05796338 |
| Sri Lanka | 1.01740466 |
| Nepal | 1.03613494 |

1. The eliminated overall performance

| Country | Criteria | | | | | | | | | | | | | |
| --- | --- | --- | --- | --- | --- | --- | --- | --- | --- | --- | --- | --- | --- | --- |
|  | A1 | A2 | A3 | A4 | A5 | A6 | A7 | A8 | A9 | A10 | A11 | A12 | A13 | A14 |
| Iraq | 0.935 | 0.960 | 0.999 | 0.903 | 0.944 | 0.954 | 0.996 | 0.930 | 0.930 | 1.000 | 0.966 | 0.941 | 0.985 | 0.907 |
| Turkey | 0.932 | 0.964 | 1.010 | 0.996 | 1.007 | 0.973 | 1.000 | 0.918 | 0.976 | 1.006 | 0.969 | 0.942 | 0.951 | 0.957 |
| Lebanon | 0.868 | 0.885 | 0.903 | 0.918 | 0.918 | 0.879 | 0.891 | 0.895 | 0.840 | 0.911 | 0.918 | 0.918 | 0.696 | 0.771 |
| Israel | 1.133 | 1.083 | 1.163 | 1.084 | 1.087 | 1.134 | 1.162 | 1.132 | 1.109 | 1.156 | 1.133 | 1.145 | 1.050 | 1.079 |
| Saudi Arabia | 0.996 | 0.972 | 1.051 | 0.942 | 0.998 | 1.008 | 1.051 | 0.977 | 1.010 | 1.045 | 1.012 | 0.970 | 1.039 | 1.002 |
| Oman | 0.999 | 0.974 | 1.046 | 0.942 | 1.004 | 0.998 | 1.033 | 1.025 | 1.002 | 1.040 | 1.014 | 1.020 | 1.000 | 0.933 |
| UAE | 1.036 | 0.972 | 1.067 | 0.976 | 1.007 | 1.000 | 1.037 | 1.016 | 1.040 | 1.055 | 1.031 | 1.013 | 1.035 | 0.983 |
| Qatar | 0.989 | 0.871 | 0.989 | 0.890 | 0.941 | 0.930 | 0.976 | 0.966 | 0.956 | 0.977 | 0.937 | 0.951 | 0.949 | 0.871 |
| Kuwait | 0.987 | 0.907 | 0.997 | 0.898 | 0.956 | 0.947 | 0.982 | 0.974 | 0.971 | 0.994 | 0.961 | 0.961 | 0.953 | 0.889 |
| Bahrain | 1.081 | 1.002 | 1.079 | 0.983 | 1.006 | 1.016 | 1.051 | 1.081 | 1.032 | 1.069 | 1.042 | 1.066 | 0.998 | 0.937 |
| Egypt | 0.932 | 1.002 | 1.023 | 0.966 | 0.962 | 1.012 | 1.031 | 0.936 | 0.976 | 1.027 | 1.002 | 0.971 | 0.991 | 0.955 |
| India | 0.951 | 1.045 | 1.052 | 0.976 | 0.986 | 1.030 | 1.054 | 0.886 | 1.043 | 1.055 | 1.015 | 0.936 | 1.060 | 1.060 |
| Pakistan | 0.855 | 0.978 | 0.972 | 0.923 | 0.915 | 0.971 | 0.981 | 0.853 | 0.944 | 0.983 | 0.954 | 0.907 | 0.937 | 0.929 |
| Bangladesh | 0.959 | 1.040 | 1.049 | 0.978 | 0.970 | 1.042 | 1.058 | 0.940 | 1.058 | 1.055 | 1.013 | 1.008 | 0.990 | 0.971 |
| Sri Lanka | 0.922 | 0.993 | 1.009 | 0.985 | 0.988 | 0.987 | 1.013 | 0.955 | 0.952 | 1.007 | 0.968 | 1.012 | 0.876 | 0.911 |
| Nepal | 0.917 | 1.036 | 1.022 | 0.954 | 0.957 | 1.036 | 1.018 | 0.975 | 0.999 | 1.031 | 0.986 | 1.024 | 0.938 | 0.942 |

1. The removal effect matrix

| Criteria | A1 | A2 | A3 | A4 | A5 | A6 | A7 |
| --- | --- | --- | --- | --- | --- | --- | --- |
| Removal effect | 1.0443 | 0.8488 | 0.1028 | 1.2202 | 0.8886 | 0.6197 | 0.2014 |
| Criteria | A8 | A9 | A10 | A11 | A12 | A13 | A14 |
| Removal effect | 1.0754 | 0.6972 | 0.1247 | 0.6141 | 0.7500 | 1.0862 | 1.4391 |

1. The obtained criteria weight

| Criteria | A1 | A2 | A3 | A4 | A5 | A6 | A7 |
| --- | --- | --- | --- | --- | --- | --- | --- |
| Weight | 0.097 | 0.079 | 0.010 | 0.114 | 0.083 | 0.058 | 0.019 |
| Criteria | A8 | A9 | A10 | A11 | A12 | A13 | A14 |
| Weight | 0.100 | 0.065 | 0.012 | 0.057 | 0.070 | 0.101 | 0.134 |

1. The weighted aggregated performance scores for non-beneficial criteria

| Country | Non-benefit criteria | | | | $\bar{I}_{i}$ |
| --- | --- | --- | --- | --- | --- |
|  | A4 | A5 | A13 | A14 |  |
| Iraq | 7.652 | 2.477 | 47.926 | 32.756 | 90.811 |
| Turkey | 4.553 | 1.018 | 100.637 | 30.592 | 136.800 |
| Lebanon | 0 | 0 | 109.244 | 33.629 | 142.872 |
| Israel | 7.680 | 2.751 | 108.654 | 33.208 | 152.292 |
| Saudi Arabia | 7.768 | 2.506 | 47.180 | 29.334 | 86.789 |
| Oman | 7.752 | 2.368 | 93.863 | 33.508 | 137.491 |
| UAE | 7.660 | 2.572 | 79.728 | 32.679 | 122.639 |
| Qatar | 7.652 | 2.343 | 83.983 | 33.390 | 127.368 |
| Kuwait | 7.699 | 2.331 | 91.727 | 33.346 | 135.102 |
| Bahrain | 7.715 | 2.688 | 105.262 | 33.776 | 149.441 |
| Egypt | 7.242 | 2.637 | 87.103 | 32.114 | 129.095 |
| India | 7.574 | 2.676 | 0 | 0 | 10.250 |
| Pakistan | 6.967 | 2.588 | 88.309 | 29.024 | 126.888 |
| Bangladesh | 7.528 | 2.738 | 101.577 | 32.710 | 144.553 |
| Sri Lanka | 5.593 | 1.911 | 108.686 | 33.240 | 149.430 |
| Nepal | 7.528 | 2.695 | 106.576 | 32.946 | 149.745 |

1. The weighted aggregated performance scores for beneficial criteria

| Country | Beneficial Criteria | | | | | | | | | | $\bar{O}_{i}$ |
| --- | --- | --- | --- | --- | --- | --- | --- | --- | --- | --- | --- |
|  | A1 | A2 | A3 | A6 | A7 | A8 | A9 | A10 | A11 | A12 |  |
| Iraq | 0.9932 | 0.2727 | 0.0004 | 0.2646 | 0.0032 | 1.2325 | 0.8105 | 0.0001 | 0.1522 | 0.5619 | 80.561 |
| Turkey | 2.3737 | 0.5501 | 0.0038 | 0.2664 | 0.0195 | 4.0028 | 0.2640 | 0.0070 | 0.3176 | 1.1512 | 126.551 |
| Lebanon | 0.4381 | 0.1660 | 0.0066 | 0.1648 | 0.0287 | 0.1221 | 0.8279 | 0.0031 | 0 | 0 | 132.623 |
| Israel | 0.3849 | 3.1612 | 0.0027 | 0.2152 | 0.0070 | 0.4205 | 0.8210 | 0.0094 | 0.2237 | 0.1323 | 142.042 |
| Saudi Arabia | 0.8228 | 1.7251 | 0.0010 | 0.2837 | 0.0021 | 1.7962 | 0.2935 | 0.0049 | 0.2344 | 1.6534 | 76.539 |
| Oman | 0.6066 | 1.4062 | 0.0017 | 0.3916 | 0.0184 | 0.1670 | 0.3687 | 0.0058 | 0.1824 | 0.1599 | 127.241 |
| UAE | 0.2521 | 3.1077 | 0.0003 | 0.7629 | 0.0448 | 0.6837 | 0.1323 | 0.0077 | 0.1864 | 0.5344 | 112.389 |
| Qatar | 0 | 5.1405 | 0 | 0.4462 | 0.0119 | 0.1384 | 0.1535 | 0.0066 | 0.3350 | 0.2193 | 117.118 |
| Kuwait | 0.0775 | 2.4838 | 0.0021 | 0.3981 | 0.0216 | 0.1880 | 0.1458 | 0.0045 | 0.2103 | 0.2587 | 124.852 |
| Bahrain | 0.0011 | 1.7083 | 0.0007 | 0.7092 | 0.0443 | 0 | 0.3998 | 0.0071 | 0.2162 | 0.0610 | 139.191 |
| Egypt | 4.0893 | 0.1754 | 0.0040 | 0.0712 | 0.0009 | 3.6141 | 0.5052 | 0.0025 | 0.1247 | 0.6550 | 118.845 |
| India | 6.2591 | 0.0624 | 0.0034 | 0.1342 | 0.0054 | 62.3318 | 0.0605 | 0.0025 | 0.2759 | 7.6711 | 0 |
| Pakistan | 8.4135 | 0.0154 | 0.0046 | 0.0317 | 0.0009 | 9.3042 | 0.1982 | 0 | 0.1046 | 0.9859 | 116.638 |
| Bangladesh | 4.1933 | 0.0801 | 0.0040 | 0.0524 | 0 | 8.7738 | 0 | 0.0017 | 0.2854 | 0.4296 | 134.303 |
| Sri Lanka | 3.2491 | 0.1196 | 0.0036 | 0.1260 | 0.0037 | 0.9382 | 0.6829 | 0.0055 | 0.3105 | 0.0149 | 139.180 |
| Nepal | 7.9584 | 0 | 0.0072 | 0 | 0.0196 | 0.9366 | 0.2139 | 0.0028 | 0.3429 | 0.0409 | 139.495 |

1. The Linear transformation of non-beneficial and beneficial scores

| Country | $I_{i}$ | $O_{i}$ |
| --- | --- | --- |
| Iraq | 80.561 | 3.691 |
| Turkey | 126.551 | 1.139 |
| Lebanon | 132.623 | 0.230 |
| Israel | 142.042 | 0.586 |
| Saudi Arabia | 76.539 | 1.586 |
| Oman | 127.241 | 0.116 |
| UAE | 112.389 | 0.260 |
| Qatar | 117.118 | 0.114 |
| Kuwait | 124.852 | 0.019 |
| Bahrain | 139.191 | 0.083 |
| Egypt | 118.845 | 0.687 |
| India | 0 | 7.409 |
| Pakistan | 116.638 | 0.688 |
| Bangladesh | 134.303 | 0.116 |
| Sri Lanka | 139.180 | 0.413 |
| Nepal | 139.495 | 0 |

1. The final evaluation score of studied countries in SREB

| Country | Final evaluation score |
| --- | --- |
| Iraq | 76.843 |
| Turkey | 120.280 |
| Lebanon | 125.444 |
| Israel | 135.218 |
| Saudi Arabia | 70.715 |
| Oman | 119.948 |
| United Arab Emirates | 105.239 |
| Qatar | 109.822 |
| Kuwait | 117.462 |
| Bahrain | 131.865 |
| Egypt | 112.123 |
| India | 0.000 |
| Pakistan | 109.917 |
| Bangladesh | 127.010 |
| Sri Lanka | 132.184 |
| Nepal | 132.086 |

1. TOPSIS normalized matrix

| **Country** | **A1** | **A2** | **A3** | **A4** | **A5** | **A6** | **A7** | **A8** | **A9** | **A10** | **A11** | **A12** | **A13** | **A14** |
| --- | --- | --- | --- | --- | --- | --- | --- | --- | --- | --- | --- | --- | --- | --- |
| Iraq | 0.118 | 0.053 | 0.053 | 0.015 | 0.099 | 0.347 | 0.071 | 0.003 | 0.978 | 0.000 | 0.444 | 0.073 | 0.561 | 0.030 |
| Turkey | 0.282 | 0.107 | 0.523 | 0.414 | 0.630 | 0.349 | 0.436 | 0.430 | 0.319 | 0.750 | 0.926 | 0.150 | 0.079 | 0.094 |
| Lebanon | 0.052 | 0.032 | 0.910 | 1.000 | 1.000 | 0.216 | 0.641 | 0.013 | 1.000 | 0.344 | 0.000 | 0.000 | 0.000 | 0.004 |
| Israel | 0.046 | 0.615 | 0.381 | 0.011 | 0.000 | 0.282 | 0.155 | 0.045 | 0.993 | 1.000 | 0.652 | 0.017 | 0.005 | 0.017 |
| Saudi Arabia | 0.098 | 0.336 | 0.143 | 0.000 | 0.089 | 0.372 | 0.047 | 0.193 | 0.354 | 0.531 | 0.684 | 0.216 | 0.568 | 0.131 |
| Oman | 0.072 | 0.274 | 0.242 | 0.002 | 0.139 | 0.513 | 0.412 | 0.018 | 0.445 | 0.625 | 0.532 | 0.021 | 0.141 | 0.008 |
| UAE | 0.030 | 0.605 | 0.039 | 0.014 | 0.065 | 1.000 | 1.000 | 0.074 | 0.160 | 0.813 | 0.544 | 0.070 | 0.270 | 0.032 |
| Qatar | 0.000 | 1.000 | 0.000 | 0.015 | 0.148 | 0.585 | 0.266 | 0.015 | 0.184 | 0.719 | 0.977 | 0.029 | 0.231 | 0.011 |
| Kuwait | 0.010 | 0.483 | 0.290 | 0.009 | 0.153 | 0.522 | 0.482 | 0.020 | 0.177 | 0.469 | 0.613 | 0.034 | 0.160 | 0.013 |
| Bahrain | 0.000 | 0.332 | 0.094 | 0.007 | 0.023 | 0.930 | 0.990 | 0.000 | 0.484 | 0.750 | 0.631 | 0.008 | 0.036 | 0.000 |
| Egypt | 0.486 | 0.034 | 0.560 | 0.068 | 0.041 | 0.093 | 0.020 | 0.388 | 0.609 | 0.250 | 0.364 | 0.085 | 0.203 | 0.049 |
| India | 0.744 | 0.012 | 0.478 | 0.025 | 0.027 | 0.176 | 0.121 | 0.660 | 0.074 | 0.250 | 0.805 | 1.000 | 1.000 | 1.000 |
| Pakistan | 1.000 | 0.003 | 0.641 | 0.103 | 0.059 | 0.042 | 0.019 | 1.000 | 0.238 | 0.000 | 0.305 | 0.129 | 0.192 | 0.141 |
| Bangladesh | 0.499 | 0.016 | 0.560 | 0.031 | 0.005 | 0.069 | 0.000 | 0.943 | 0.000 | 0.156 | 0.833 | 0.056 | 0.070 | 0.351 |
| Sri Lanka | 0.386 | 0.023 | 0.502 | 0.280 | 0.305 | 0.165 | 0.083 | 0.101 | 0.826 | 0.594 | 0.905 | 0.002 | 0.005 | 0.016 |
| Nepal | 0.946 | 0.000 | 1.000 | 0.031 | 0.020 | 0.000 | 0.437 | 0.101 | 0.258 | 0.281 | 1.000 | 0.005 | 0.024 | 0.024 |

1. TOPSIS Weighted normalized matrix

| **Country** | **A1** | **A2** | **A3** | **A4** | **A5** | **A6** | **A7** | **A8** | **A9** | **A10** | **A11** | **A12** | **A13** | **A14** |
| --- | --- | --- | --- | --- | --- | --- | --- | --- | --- | --- | --- | --- | --- | --- |
| Iraq | 0.012 | 0.004 | 0.001 | 0.002 | 0.008 | 0.020 | 0.001 | 0.000 | 0.064 | 0.000 | 0.025 | 0.005 | 0.057 | 0.004 |
| Turkey | 0.028 | 0.008 | 0.005 | 0.047 | 0.052 | 0.020 | 0.008 | 0.043 | 0.021 | 0.009 | 0.053 | 0.011 | 0.008 | 0.013 |
| Lebanon | 0.005 | 0.003 | 0.009 | 0.114 | 0.083 | 0.013 | 0.012 | 0.001 | 0.065 | 0.004 | 0.000 | 0.000 | 0.000 | 0.001 |
| Israel | 0.004 | 0.049 | 0.004 | 0.001 | 0.000 | 0.016 | 0.003 | 0.005 | 0.065 | 0.012 | 0.037 | 0.001 | 0.001 | 0.002 |
| Saudi Arabia | 0.010 | 0.027 | 0.001 | 0.000 | 0.007 | 0.022 | 0.001 | 0.019 | 0.023 | 0.006 | 0.039 | 0.015 | 0.058 | 0.018 |
| Oman | 0.007 | 0.022 | 0.002 | 0.000 | 0.012 | 0.030 | 0.008 | 0.002 | 0.029 | 0.007 | 0.030 | 0.001 | 0.014 | 0.001 |
| UAE | 0.003 | 0.048 | 0.000 | 0.002 | 0.005 | 0.058 | 0.019 | 0.007 | 0.010 | 0.009 | 0.031 | 0.005 | 0.027 | 0.004 |
| Qatar | 0.000 | 0.079 | 0.000 | 0.002 | 0.012 | 0.034 | 0.005 | 0.001 | 0.012 | 0.008 | 0.056 | 0.002 | 0.023 | 0.002 |
| Kuwait | 0.001 | 0.038 | 0.003 | 0.001 | 0.013 | 0.030 | 0.009 | 0.002 | 0.012 | 0.005 | 0.035 | 0.002 | 0.016 | 0.002 |
| Bahrain | 0.000 | 0.026 | 0.001 | 0.001 | 0.002 | 0.054 | 0.019 | 0.000 | 0.032 | 0.009 | 0.036 | 0.001 | 0.004 | 0.000 |
| Egypt | 0.047 | 0.003 | 0.005 | 0.008 | 0.003 | 0.005 | 0.000 | 0.039 | 0.040 | 0.003 | 0.021 | 0.006 | 0.021 | 0.007 |
| India | 0.073 | 0.001 | 0.005 | 0.003 | 0.002 | 0.010 | 0.002 | 0.066 | 0.005 | 0.003 | 0.046 | 0.070 | 0.101 | 0.134 |
| Pakistan | 0.097 | 0.000 | 0.006 | 0.012 | 0.005 | 0.002 | 0.000 | 0.100 | 0.016 | 0.000 | 0.017 | 0.009 | 0.019 | 0.019 |
| Bangladesh | 0.049 | 0.001 | 0.005 | 0.004 | 0.000 | 0.004 | 0.000 | 0.095 | 0.000 | 0.002 | 0.048 | 0.004 | 0.007 | 0.047 |
| Sri Lanka | 0.038 | 0.002 | 0.005 | 0.032 | 0.025 | 0.010 | 0.002 | 0.010 | 0.054 | 0.007 | 0.052 | 0.000 | 0.001 | 0.002 |
| Nepal | 0.092 | 0.000 | 0.010 | 0.004 | 0.002 | 0.000 | 0.008 | 0.010 | 0.017 | 0.003 | 0.057 | 0.000 | 0.002 | 0.003 |

1. TOPSIS final score

| **Country** | **D+** | **D-** | **TOPSIS Score** |
| --- | --- | --- | --- |
| Iraq | 0.183 | 0.206 | 0.529 |
| Turkey | 0.159 | 0.189 | 0.542 |
| Lebanon | 0.233 | 0.181 | 0.437 |
| Israel | 0.161 | 0.236 | 0.594 |
| Saudi Arabia | 0.166 | 0.195 | 0.541 |
| Oman | 0.171 | 0.216 | 0.558 |
| UAE | 0.165 | 0.220 | 0.570 |
| Qatar | 0.168 | 0.228 | 0.576 |
| Kuwait | 0.173 | 0.215 | 0.554 |
| Bahrain | 0.170 | 0.231 | 0.576 |
| Egypt | 0.148 | 0.215 | 0.592 |
| India | 0.207 | 0.189 | 0.478 |
| Pakistan | 0.136 | 0.238 | 0.637 |
| Bangladesh | 0.151 | 0.222 | 0.595 |
| Sri Lanka | 0.165 | 0.212 | 0.563 |
| Nepal | 0.159 | 0.241 | 0.603 |

1. EDAS Positive distance to average solution

| **Country** | **A1** | **A2** | **A3** | **A4** | **A5** | **A6** | **A7** | **A8** | **A9** | **A10** | **A11** | **A12** | **A13** | **A14** |
| --- | --- | --- | --- | --- | --- | --- | --- | --- | --- | --- | --- | --- | --- | --- |
| Iraq | 0.000 | 0.000 | 0.000 | 0.791 | 0.369 | 0.000 | 0.000 | 0.000 | 1.023 | 0.000 | 0.000 | 0.000 | 0.000 | 0.727 |
| Turkey | 0.000 | 0.000 | 0.070 | 0.000 | 0.000 | 0.000 | 0.151 | 0.689 | 0.000 | 0.159 | 0.357 | 0.249 | 0.642 | 0.210 |
| Lebanon | 0.000 | 0.000 | 0.293 | 0.000 | 0.000 | 0.000 | 0.427 | 0.000 | 1.065 | 0.000 | 0.000 | 0.000 | 0.996 | 0.933 |
| Israel | 0.000 | 1.418 | 0.000 | 0.816 | 0.853 | 0.000 | 0.000 | 0.000 | 1.051 | 0.302 | 0.017 | 0.000 | 0.972 | 0.831 |
| Saudi Arabia | 0.000 | 0.346 | 0.000 | 0.896 | 0.421 | 0.042 | 0.000 | 0.000 | 0.000 | 0.035 | 0.056 | 0.762 | 0.000 | 0.000 |
| Oman | 0.000 | 0.108 | 0.000 | 0.882 | 0.176 | 0.371 | 0.118 | 0.000 | 0.002 | 0.088 | 0.000 | 0.000 | 0.363 | 0.903 |
| UAE | 0.000 | 1.378 | 0.000 | 0.797 | 0.537 | 1.504 | 0.909 | 0.000 | 0.000 | 0.195 | 0.000 | 0.000 | 0.000 | 0.708 |
| Qatar | 0.000 | 2.895 | 0.000 | 0.790 | 0.131 | 0.538 | 0.000 | 0.000 | 0.000 | 0.142 | 0.420 | 0.000 | 0.000 | 0.876 |
| Kuwait | 0.000 | 0.912 | 0.000 | 0.833 | 0.110 | 0.391 | 0.213 | 0.000 | 0.000 | 0.000 | 0.000 | 0.000 | 0.275 | 0.865 |
| Bahrain | 0.000 | 0.334 | 0.000 | 0.848 | 0.742 | 1.340 | 0.896 | 0.000 | 0.077 | 0.159 | 0.000 | 0.000 | 0.832 | 0.966 |
| Egypt | 0.608 | 0.000 | 0.092 | 0.417 | 0.651 | 0.000 | 0.000 | 0.529 | 0.317 | 0.000 | 0.000 | 0.000 | 0.085 | 0.573 |
| India | 1.441 | 0.000 | 0.044 | 0.719 | 0.722 | 0.000 | 0.000 | 1.570 | 0.000 | 0.000 | 0.207 | 6.914 | 0.000 | 0.000 |
| Pakistan | 2.268 | 0.000 | 0.138 | 0.166 | 0.565 | 0.000 | 0.000 | 2.872 | 0.000 | 0.000 | 0.000 | 0.080 | 0.135 | 0.000 |
| Bangladesh | 0.648 | 0.000 | 0.092 | 0.677 | 0.831 | 0.000 | 0.000 | 2.653 | 0.000 | 0.000 | 0.241 | 0.000 | 0.680 | 0.000 |
| Sri Lanka | 0.285 | 0.000 | 0.058 | 0.000 | 0.000 | 0.000 | 0.000 | 0.000 | 0.731 | 0.070 | 0.332 | 0.000 | 0.973 | 0.839 |
| Nepal | 2.093 | 0.000 | 0.345 | 0.677 | 0.755 | 0.000 | 0.152 | 0.000 | 0.000 | 0.000 | 0.449 | 0.000 | 0.886 | 0.771 |

1. EDAS negative distance to average solution

| **Country** | **A1** | **A2** | **A3** | **A4** | **A5** | **A6** | **A7** | **A8** | **A9** | **A10** | **A11** | **A12** | **A13** | **A14** |
| --- | --- | --- | --- | --- | --- | --- | --- | --- | --- | --- | --- | --- | --- | --- |
| Iraq | 0.581 | 0.737 | 0.201 | 0.000 | 0.000 | 0.016 | 0.340 | 0.946 | 0.000 | 0.269 | 0.241 | 0.354 | 1.526 | 0.000 |
| Turkey | 0.051 | 0.530 | 0.000 | 2.035 | 2.214 | 0.011 | 0.000 | 0.000 | 0.238 | 0.000 | 0.000 | 0.000 | 0.000 | 0.000 |
| Lebanon | 0.794 | 0.817 | 0.000 | 6.186 | 4.016 | 0.320 | 0.000 | 0.908 | 0.000 | 0.072 | 0.792 | 0.928 | 0.000 | 0.000 |
| Israel | 0.815 | 0.000 | 0.012 | 0.000 | 0.000 | 0.167 | 0.227 | 0.786 | 0.000 | 0.000 | 0.000 | 0.793 | 0.000 | 0.000 |
| Saudi Arabia | 0.646 | 0.000 | 0.149 | 0.000 | 0.000 | 0.000 | 0.373 | 0.219 | 0.172 | 0.000 | 0.000 | 0.000 | 1.556 | 0.090 |
| Oman | 0.730 | 0.000 | 0.092 | 0.000 | 0.000 | 0.000 | 0.000 | 0.890 | 0.000 | 0.000 | 0.132 | 0.765 | 0.000 | 0.000 |
| UAE | 0.866 | 0.000 | 0.209 | 0.000 | 0.000 | 0.000 | 0.000 | 0.677 | 0.544 | 0.000 | 0.118 | 0.382 | 0.218 | 0.000 |
| Qatar | 0.963 | 0.000 | 0.231 | 0.000 | 0.000 | 0.000 | 0.078 | 0.902 | 0.497 | 0.000 | 0.000 | 0.704 | 0.043 | 0.000 |
| Kuwait | 0.932 | 0.000 | 0.064 | 0.000 | 0.000 | 0.000 | 0.000 | 0.881 | 0.511 | 0.001 | 0.031 | 0.664 | 0.000 | 0.000 |
| Bahrain | 0.962 | 0.000 | 0.177 | 0.000 | 0.000 | 0.000 | 0.000 | 0.959 | 0.000 | 0.000 | 0.010 | 0.866 | 0.000 | 0.000 |
| Egypt | 0.000 | 0.810 | 0.000 | 0.000 | 0.000 | 0.606 | 0.408 | 0.000 | 0.000 | 0.126 | 0.341 | 0.259 | 0.000 | 0.000 |
| India | 0.000 | 0.894 | 0.000 | 0.000 | 0.000 | 0.414 | 0.273 | 0.000 | 0.708 | 0.126 | 0.000 | 0.000 | 3.497 | 7.084 |
| Pakistan | 0.000 | 0.929 | 0.000 | 0.000 | 0.000 | 0.727 | 0.410 | 0.000 | 0.393 | 0.269 | 0.414 | 0.000 | 0.000 | 0.165 |
| Bangladesh | 0.000 | 0.881 | 0.000 | 0.000 | 0.000 | 0.664 | 0.435 | 0.000 | 0.849 | 0.179 | 0.000 | 0.489 | 0.000 | 1.862 |
| Sri Lanka | 0.000 | 0.852 | 0.000 | 1.087 | 0.633 | 0.439 | 0.323 | 0.573 | 0.000 | 0.000 | 0.000 | 0.913 | 0.000 | 0.000 |
| Nepal | 0.000 | 0.941 | 0.000 | 0.000 | 0.000 | 0.824 | 0.000 | 0.573 | 0.355 | 0.108 | 0.000 | 0.887 | 0.000 | 0.000 |

1. EDAS final score

| **Country** | **SP** | **SN** | **EDAS Score** |
| --- | --- | --- | --- |
| Iraq | 0.285 | 0.416 | 0.509 |
| Turkey | 0.206 | 0.479 | 0.445 |
| Lebanon | 0.306 | 1.401 | 0.182 |
| Israel | 0.559 | 0.228 | 0.720 |
| Saudi Arabia | 0.224 | 0.275 | 0.525 |
| Oman | 0.307 | 0.223 | 0.587 |
| UAE | 0.446 | 0.245 | 0.654 |
| Qatar | 0.505 | 0.274 | 0.675 |
| Kuwait | 0.347 | 0.262 | 0.595 |
| Bahrain | 0.500 | 0.253 | 0.680 |
| Egypt | 0.321 | 0.146 | 0.621 |
| India | 0.936 | 1.454 | 0.500 |
| Pakistan | 0.596 | 0.198 | 0.750 |
| Bangladesh | 0.559 | 0.458 | 0.641 |
| Sri Lanka | 0.307 | 0.397 | 0.528 |
| Nepal | 0.569 | 0.266 | 0.712 |

1. Ranking comparison

| Country | MEREC-OCRA | MEREC-TOPSIS | MEREC-EDAS |
| --- | --- | --- | --- |
| Iraq | 14 | 14 | 13 |
| Turkey | 7 | 12 | 15 |
| Lebanon | 6 | 16 | 16 |
| Israel | 1 | 4 | 2 |
| Saudi Arabia | 15 | 13 | 12 |
| Oman | 8 | 10 | 10 |
| United Arab Emirates | 13 | 8 | 6 |
| Qatar | 12 | 7 | 5 |
| Kuwait | 9 | 11 | 9 |
| Bahrain | 4 | 6 | 4 |
| Egypt | 10 | 5 | 8 |
| India | 16 | 15 | 14 |
| Pakistan | 11 | 1 | 1 |
| Bangladesh | 5 | 3 | 7 |
| Sri Lanka | 2 | 9 | 11 |
| Nepal | 3 | 2 | 3 |
